# Dosimetric effects near implanted vascular access ports: an examination of external photon beam dose calculations

**DOI:** 10.1120/jacmp.v10i3.2886

**Published:** 2009-05-28

**Authors:** Michael S. Gossman, Jan P. Seuntjens, Monica F. Serban, Kelly J. Christian, Raymond C. Lawson, Mary A. Robertson, Jeffrey P. Lopez, Terry E. Justice

**Affiliations:** ^1^ Tri‐State Regional Cancer Center Ashland Kentucky 41101 USA; ^2^ McGill University Montreal General Hospital Medical Physics Unit Montreal QC H3G 1A4 Canada; ^3^ Maisonneuve‐Rosemont Hospital Radiation Oncology Department Montreal QC H1T 2M4 Canada; ^4^ Bard Access Systems, Inc. Salt Lake City Utah 84116 USA

**Keywords:** AAA, attenuation, backscatter, lateral scatter, Monte Carlo, PBC, vascular port

## Abstract

Vascular access ports are used widely in the administering of drugs for radiation oncology patients. Their dosimetric effect on radiation therapy delivery in photon beams has not been rigorously established. In this work, the effects on external beam fields when any of a variety of vascular access ports were included in the path of a high energy beam are studied. This study specifically identifies sidescatter and backscatter consequences as well as attenuation effects. The study was divided into two parts. First, a total of 18 ports underwent extended HU range CT scanning followed by 3D computer treatment planning, where homogeneous and heterogeneous plans were created for photon beams of energy 6 MV and 18 MV using a Pencil Beam Convolution (PBC) algorithm. Dose points were analyzed at locations around each device. A total of 1,440 points were reviewed in this section of the study. A replicate of the largest vascular access port was created in the treatment planning workspace for further investigation with alternative treatment planning algorithms. Then, plans were generated identical to the above and compared to the results of dose computation between the Pencil Beam Convolution algorithm, the Analytical Anisotropic Algorithm (AAA), and the EGSnrc Monte Carlo algorithm with user code DOSRZnrc (MC). A total of 300 points were reviewed in this part of the study. It was concluded that ports with more bulky construction and those with partial metal composition create the largest changes. Similar effects were observed for similar port configurations. Considerable differences between the PBC and AAA in comparison to MC are noted and discussed. By thorough examination of planning system results, the presented vascular access ports may now be ranked according to the greatest amount of change exhibited within a treatment planning system. Effects of backscatter, lateral scatter, and attenuation are up to 5.0%, 3.4% and 16.8% for 6 MV and 7.0%, 7.7% and 7.2% for 18 MV, respectively.

PACS numbers: 87.56.bd, 61.82.Bg, 41.50.+h, 87.19.Hh, 87.55.Gh, 87.55.K−, 87.55.N−, 87.55.−x, 81.40.Wx, 87.55.−x, 87.53.Bn, and 87.59.bd

## I. INTRODUCTION

Vascular access ports have been widely used in the care of oncology patients for several decades. The first documentation of use is as early as January of 1969.^(^ ^1^ ^,^ ^2^ ^)^ The purpose of the device is to supply intravenous chemotherapeutic agents, antibiotics and other drugs on a regular basis.

This obviates the need for frequent venipunctures and maintenance of peripheral lines. Having a port in place also makes obtaining blood samples easier for the same reason. Over the years, the design of vascular access ports has changed, just as other intravenous line management devices have evolved.^(3)^

**Figure 1:**
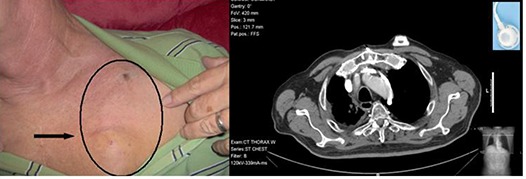
(a) (left): A photographic illustration of the distention of skin overlaying MRI Plastic Lumen port model 0602660 after placement is shown. The shape of the port is easily seen; (b) (right): A CT scan of the patient's chest is shown, making the exact position of the port relative to his anatomy evident.

Port catheters are primarily placed in either the subclavian, axillary, or internal jugular veins. Typically, the catheters are tunneled from the venotomy to a place on the upper chest just inferior to the infraclavicular fossa, where the port is positioned subdermally. For example, in Fig. 1 we show the clinical presentation of the device in a patient from past treatment documentation. A patient diagnosed with non‐small cell lung cancer was prescribed a Bard Access Systems model 0602660 MRI plastic lumen port (Bard Access Systems, Inc., Salt Lake City, UT). A photograph of the patient's chest indicates the underlying placement of the access port as it distends the skin surface (Fig. 1(a)). A CT image reveals the exact placement of the port in the axial view (Fig. 1(b)).

These devices vary in construction and are available in many different shapes and sizes.^(4)^ There are single and dual injectable ports as well as recently available power injectable ports. The plastic construction materials include Delrin (polyacetal resin), silicone, polycarbonate plastic, and polyurethane. Delrin is used to make the port body. The septum and suture plugs are made from silicone. The catheter locking collar is made from polycarbonate plastics for rigidity. Additionally, some polyurethane and silicone catheters are doped with barium sulfate to make the catheter well‐observed in diagnostic radiology. These construction materials provide more stiffness than silicone, and result in smoother positioning and thus less irritation.^(^ ^5^ ^,^ ^6^ ^,^ ^7^ ^)^ The only significant scattering material identified in the construction of ports presented here is the titanium metal alloy. Identified as Ti (6A14V), it is composed of 6% aluminum and 4% vanadium; the resulting metal is very dense. No stainless steel is found in any of the Bard ports.

Of course, it is important to examine radiation dosimetric effects near foreign materials implanted in the body. Concerning titanium, Mian et al.^(8)^ found backscattering in 1.1 mm of titanium at the interface may be as much as 14% greater for 6 MV and 11% for 25 MV. Similarly for mandibular bridging plates, they observed that at 6 MV, the backscatter dose was greatest. It is noteworthy that bridging plates contain more metal than ports in general. Still, this study provided early evidence that therapy beam dose distributions should be considered, as some vascular access ports are now constructed with such metals.

Noriega et al.^(9)^ used film, and observed that the attenuation was as much as 17.5% for 6 MV and 10% for 15 MV in a very small selection of ports. Attenuation was found to decrease with increasing energy. The devices studied were very similar in design and shape.

In a third port study, 6–10 MV X rays and 5 typical clinical electron beams were used.^(10)^ Measurements were performed with a 0.6 cc parallel‐plane ionization chamber in polystyrene and also by 0.1cc micro‐ionization chambers in a water phantom. Stainless steel ports were included in the research. Although ports were submerged in a water phantom, they were not filled with saline or water to resemble the clinical scenario of having the catheter and port without air. However, this study (Bagne et al.) concluded that for X rays, a dose reduction on attenuation may be as high as 17% for stainless steel. Additionally, changes of as much as 45% were identified when using electrons for therapy at energies up to 18 MeV, passing the beam through a port. This study concluded that ports should not be placed in the path of electron beams.

However, though the dosimetry of electron beams through vascular ports is troublesome, the clinical acceptability of photon beams through these ports is debated. Studies in this area are limited. Some photon results may still be observed as acceptable clinically to radiation oncologists. Vascular access ports research, in general, is still lacking. To date, no research has been formalized for clarity on the impact of dose changes identifiable during treatment planning simulation. With various models of such devices now available, there is an explicit need to quantify the magnitude of attenuation, backscatter, and lateral scatter. A variety of calculation algorithms for treatment planning are currently available. This research addresses both the magnitude of effects on dosimetry, while providing a direct comparison to results from the various planning systems.

The scope of this study is to characterize the effect of the Bard Access Systems ports on external beam radiation therapy. Moreover, the study allows for the determination as to whether there is a difference between similar port designs affecting radiation treatment regimens, and to discuss whether or not port placement alternatives or radiation therapy should be avoided.^(^ ^11^ ^,^ ^12^ ^,^ ^13^ ^)^ The magnitude of dosimetric effects in treatment planning, by placing various vascular ports in the beam, are yet to be published.

The dosimetric differences between treatment plans designed for each individual vascular access port and standard plans without such ports were determined. This was conducted for all representative port models available from the primary vascular access port marketing company. The present research encompasses results for 70% of all vascular access ports used in medicine in the United States.^(^ ^14^ ^,^ ^15^ ^,^ ^16^ ^)^

## II. MATERIALS AND METHODS

### A. Eighteen vascular access port simulations from CT: PBC calculations

Dosimetric effects of a total of 18 ports were analyzed. They represent all types of Bard Access Systems, Inc. ports manufactured today. Bard Access Systems has a 70% market share according to the latest data available from IMS Health.^(14)^ In order to plan identically for each port, all ports were given an independent CT scan under an identical geometry. The manual Water Phantom WP‐3040 (CNMC Company, Inc. Nashville, TN) defined the scanning area of interest. The dimensions of the phantom are 30cmwide×40cmlong×38cmhigh. The phantom is constructed of 3/8 inch clear acrylic on each of the sides including the white‐papered acrylic bottom.

Acrylic slabs of dimension of 24.75cmwide×24.75cmlong×8.0cmhigh were placed at the bottom of the tank. This was done to ensure that each port lay fat on a hard surface, while maintaining the position of the port for adequate lateral and backscatter build‐up equilibrium. Water was then used to fill the tank to achieve an appropriate planning depth, enabling anterior dose buildup equilibrium.

In a vascular procedure, the physician would ensure that all air is flushed from the port and infusion set prior to it being introduced into the patient. This is typically done with a saline solution. In this study, water was used to completely remove air from the system. This measure was taken for two reasons: to eliminate the effects of possible reduced attenuation as a result of a possible air‐gap, and to maintain similar association to clinical use. Since the relative electron density is identical, no change in attenuation or scattering due to the replacement of saline by water will be seen. Also, the watertight vacuum resembles the saline‐tight vacuum required to protect the patient from gases in the bloodstream. Gas removal and water replacement was conducted out of the tank first. By piercing the septum with a syringe (as is done clinically), it is readily observed that gas is removed when the flow of water from the attached sleeved catheter is stable. This also allows for a quick visible quality assurance regarding the integrity of the device. Continuing to apply injection pressure with the syringe, the vascular access port and catheter were then each submerged prior to release. Each port was lowered to a depth of 5 cm in water, at the anterior surface of the acrylic phantom, where it was then strapped down using medical adhesive tape. A MicroIntroducer Kit model 7707540, Percutaneous Introducer System model 06077800, and Winged Injection Set 0604220 (all from Bard Access Systems, Inc.) were used as needed to prime each port. Approximately 5 ml of water was passed through each submerged port, and pressure was relieved through the submerged sleeved catheter, to ensure all internal chambers were completely filled. Due to the varying size of each port, the posterior surface of the port is at precisely the 5 cm depth in water. The anterior surface of the port is closer to the surface, at 4 cm or less. Motion of the water was permitted to settle before image acquisition.

**Figure 2:**
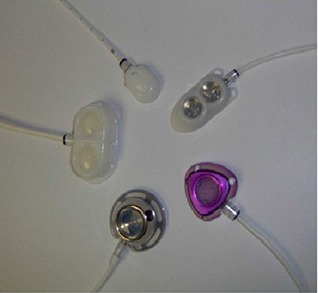
A sample selection of Bard Access ports used in this study. Starting at the 2 o'clock position, ports are identified as the Rosenblatt model 0654970, Titanium PowerPort model 1708000, Titanium Full Size port model 0605300, MRI Dual Lumen port model 0605930, and the Ultra Low Profile port model 0655640.

Each port underwent CT scanning with the model number well‐documented for data analysis (see Table 1). A photograph of a variety of vascular access ports studied is presented in Fig. 2. Computed tomography was conducted using a Lightspeed RT helical scanner (General Electric, Fairfield, CT). The protocol for stereotactic radiosurgery scanning was utilized. The average technique for the scans was 120 kVp X‐ray energy at 155 mA with a 1987 ms scan time. A 50 cm diameter circular field of view was used with a couch increment of 1.25 mm/slice. A CT scan was performed for each of the 18 ports.

**Table 1:** Monte Carlo percentage results for attenuation and scatter around vascular access port devices for (a) 6 MV and (b) 18 MV photon energies, under identical treatment planning geometry.

(a) 6MV			
	*Attenuation*	*Backscatter*	*Lateral Scatter*
TITANIUM POWERPORT (1708000)	−16.8	5.0	3.4
TITANIUM FULL SIZE PORT (0605300)	−15.4	4.6	3.1
TITANIUM X‐PORT ISP (7708540)	−14.1	4.2	2.8
TITANIUM POWERPORT ISP (1708060)	−14.1	4.2	2.8
SLIMPORT (0605560)	−13.0	3.9	2.6
LOW PROFILE TITANIUM PORT (0605490)	−10.6	3.2	2.2
ROSENBLATT (0654970)	−9.3	2.8	1.9
X‐PORT (0605840)	−8.6	2.6	1.7
DOME TITANIUM (0602870)	−8.2	2.4	1.7
MRI POWERPORT (1808000)	−4.1	1.2	0.8
X‐PORT ISP MRI (7707540)	−2.7	0.8	0.6
X‐PORT ISP (0657500)	−1.7	0.5	0.3
ULTRA LOW PROFILE PORT (0655640)	−1.7	0.5	0.3
X‐PORT DUO (0607650)	−0.7	0.2	0.1
MRI DUAL LUMEN PORT (0605930)	−0.7	0.2	0.1
LOW PROFILE MRI PORT (0603880)	0.0	0.0	0.0
PLASTIC HARD BASE PORT (0604520)	0.0	0.0	0.0
MRI FULL SIZE PORT (0605420)	0.0	0.0	0.0
(b) 18 MV			
	*Attenuation*	*Backscatter*	*Lateral Scatter*
TITANIUM POWERPORT (1708000)	−7.2	7.0	7.7
TITANIUM FULL SIZE PORT (0605300)	−6.6	6.4	7.0
TITANIUM POWERPORT ISP (1708060)	−6.2	6.1	6.7
TITANIUM X‐PORT ISP (7708540)	−6.1	5.9	6.5
SLIMPORT (0605560)	−5.9	5.8	6.3
LOW PROFILE TITANIUM PORT (0605490)	−4.6	4.5	5.0
ROSENBLATT (0654970)	−3.8	3.7	4.1
X‐PORT (0605840)	−3.5	3.4	3.8
DOME TITANIUM (0602870)	−3.5	3.4	3.8
MRI POWERPORT (1808000)	−1.8	1.7	1.9
X‐PORT ISP MRI (7707540)	−1.1	1.1	1.2
ULTRA LOW PROFILE PORT (0655640)	−0.8	0.8	0.9
X‐PORT ISP (0657500)	−0.6	0.6	0.7
X‐PORT DUO (0607650)	−0.3	0.3	0.3
MRI DUAL LUMEN PORT (0605930)	−0.3	0.3	0.3
LOW PROFILE MRI PORT (0603880)	0.0	0.0	0.0
PLASTIC HARD BASE PORT (0604520)	0.0	0.0	0.0
MRI FULL SIZE PORT (0605420)	0.0	0.0	0.0

It is important to note that the GE Lightspeed RT CT scanner was commissioned for use of the extended CT number range as recommended in studies on treatment planning commissioning with high density material considerations.^(^ ^17^ ^,^ ^18^ ^)^ This permits a CT range from −31,743 to +31,743 Hounsfield units (HU). This differs greatly from its default range of −1,024 to +3,071HU. With a net density of $4.42g/{\text{cm}}^{3}$ for that of the titanium [Ti(6A14V)] metal, the heaviest alloy used in these ports, the electron density is $12.237\times 10^{23}\;{\text{cm}}^{-3}$, which results in a relative electron density of 3.664 as compared to water. Therefore, a value of well over +3,071HU is expected. It was seen from scan acquisition that the maximum HU value for ports containing titanium was 8,315 HU. This is consistent with previously published literature for alternative alloys of titanium.^(17)^ It is important to have the correct HU value to represent the material, since it is directly involved in the dose computational software. Once the data for each port are reconstructed, the scan sets containing a total of 6,216 slices were transferred to treatment planning computers.

Varian Medical Systems, Inc. (Palo Alto, CA) model Eclipse External Beam Planning Software 6.5, Application Build 7.3.10, Photon Pencil Beam Convolution Algorithm Version PBC7310 fulfills the treatment planning requirements in this first part of the work.^(18)^ The PBC algorithm was commissioned for the Varian 21EX accelerator with photon energies of 6 MV and 18 MV and an output of 1.000 cGy/MU at the source‐to‐axis distance. The output calibration was performed according to the Task Group No. 51 protocol from the American Association of Physicists in Medicine (AAPM).^(19)^ Homogeneous and heterogeneous treatment plans were used in this study. A single anterior field was chosen, with gantry angle 180°, collimator angle 180°, and couch angle 180° in accordance with the IEC 61217 (formerly IEC 1217) coordinate system. A dose of 200 cGy at 400 MU/min was prescribed to a reference point located within the field but away from the port at 5 cm off‐axis and at 3.5 cm depth in the water. This provides adequate buildup for 6 MV and 18 MV, while also prohibiting scatter contributions which may occur if the point was positioned too close to the port. The maximum field size available on the accelerator was $40\times 40\;{\text{cm}}^{2}$. The smallest possible dose calculation grid of 1.25 mm was used.

**Figure 3:**
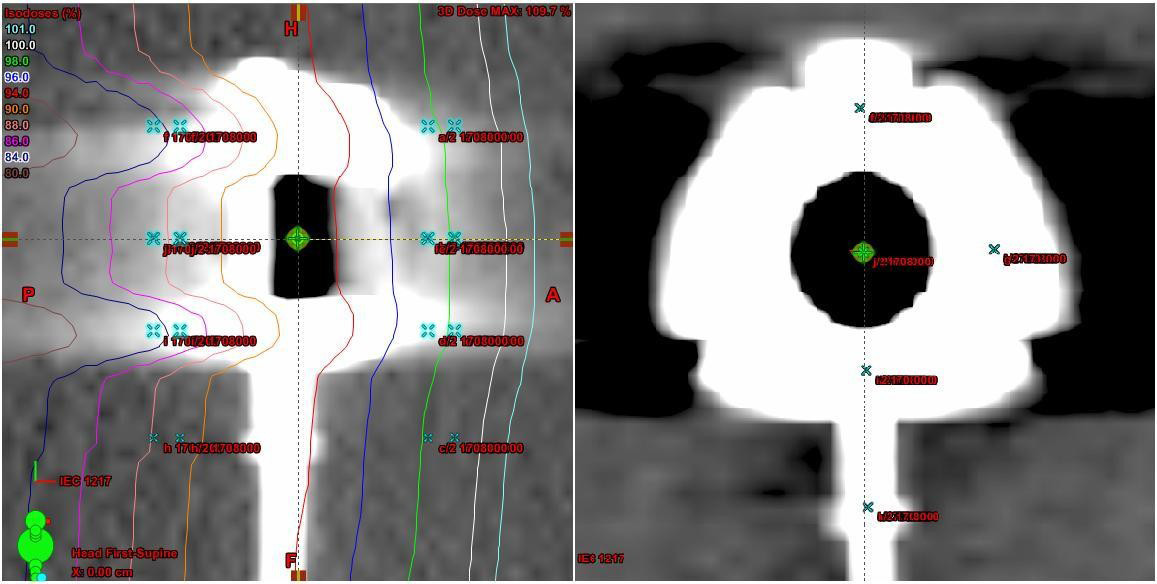
(a) (left): From the Varian Eclipse treatment planning system, points on are shown in a sagittal view placed at 2.5 mm and 5.0 mm planes anterior and posterior to the port. The isodose lines indicate the heterogeneity effect of having Bard Access port model 1708000 in the field of a 6MV beam; (b) (right): Points are seen positioned directly underneath structurally interesting areas of the port in the coronal view.

The Varian Eclipse software allows the user to place calculation points of interest on any plane of the 3D scan. A series of points were placed at planes 2.5 mm and 5 mm both anterior and posterior to the surface of each port. Five calculation points were placed in each plane. Point placement was determined based on vascular access port construction knowledge. Care was taken to place points directly underneath known areas of high atomic number, where the largest dose gradients were expected (Fig. 3(a)). The isodose lines indicate the heterogeneity effect of the Bard Access port model 1708000 in the field of a 6 MV beam. Figure 3(b) is the coronal view showing the calculation points directly positioned underneath high density areas of the port.

In order to account for the effect of the port in the beam, the software was first programmed to use the heterogeneity correction and analyzed for all density differences. The plan was copied and recalculated using no heterogeneity correction, where the port is treated as being composed only of water, as suggested for any device to be simulated by AAPM Task Group No. 63.^(20)^ The Modified Batho Power Law method was chosen as the applied correction calculation method.^(^ ^21^ ^,^ ^22^ ^)^ This heterogeneity correction algorithm is commonly used in clinics today. It has also been well described in literature to accurately simulate dose for fields that are not large and for non‐dense inhomogeneities. It was our aim to work this algorithm and present the limitations of its use when characterizing effects from metallic vascular access ports. These are intercompared with AAA and Monte Carlo later.

Heterogeneous and homogenous plans for 6 MV and 18 MV were conducted for all 18 vascular access ports using 18 different CT scans with a total of 20 calculation points each. A total of 1,440 calculation points were analyzed. The quotient of point‐dose for the two different types of plans yielded the effect of having the port in the beam.

### B. Vascular access port replicate simulation: PBC, AAA, and Monte Carlo calculations

The Photon Pencil Beam Convolution Algorithm (Varian Medical Systems, Inc., Palo Alto, CA) and the new Analytical Anisotropic Algorithm (AAA) version AAA8120 were used in this study. In addition, the Monte Carlo EGSnrc system was accessed to verify the accuracy of these two algorithms.^(23)^ The full Monte Carlo (MC) system was operated using code DOSRZnrc.^(24)^ Dose calculations of the MC system were performed with photon spectra of the identical Varian 21EX accelerator.

Monte Carlo electron transport was carried out with the photon transport cutoff at 1 keV and with the electron transport cutoff 10 keV. Photon transport was performed by simulating the standard photon interaction processes predominantly Compton scattering (with Klein‐Nishina, no binding effects) and pair production (with angular sampling using the leading term in the Koch & Motz angular distribution). Electron transport was performed with exact (single scattering) boundary crossing and the PRESTA‐II electron step algorithm.^(23)^

In an attempt to study the worst‐case scenario for vascular access port designs with the various treatment planning systems, a replicate of the device was created. The replicate volume was designed to have structural similarities to the port model chosen for analysis. As would be indicated later, this effort was derived from comparable dimensions of size and thickness of titanium as is found in the Bard Titanium PowerPort model 1708000. A cylindrical volume was created with a diameter of 3.0 cm and thickness 1.3 cm. Centered within it, a smaller cylindrical volume was created having a diameter of 1.6 cm. Using a Boolean planning system tool, the two cylinders were separated as individual volumes. For planning purposes, the medial cylindrical volume is treated as the septum, often manufactured in the center of a vascular access port to allow fluid injection or eradication. As such, a unity HU value was assigned to the entire volume. The larger cylindrical volume is treated as the rigid rim of the vascular access port. Studying the worst‐case scenario, the rim (port body) volume was assigned CT units of 8,315 HU. These Hounsfield units were registered at the CT scanner earlier for all titanium containing devices under extended CTU range.

The beam geometry was chosen to be identical to the setup procedures of the first study. As a consequence of Eclipse software limitations when contouring such a volume, an anterior geometry was achieved with a gantry angle of 270°, a couch angle of 270°, and with the collimator angle unchanged at 180°. With a body contour surrounding the device having unity density, a single beam was assigned to project towards it at 100 cm SSD. The top surface of the simulated device was at a depth of 5 cm. A dose of 200 cGy at 400 MU/min was prescribed to a reference point located within the field. The field size was $10\times 10\;{\text{cm}}^{2}$. Again, the smallest possible dose calculation grid of 1.25 mm was used.

**Figure 4:**
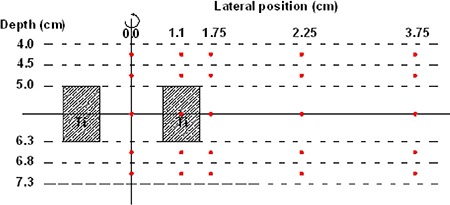
A schematic drawing showing the geometry of the model calculations (MC, AAA and PBC). The beam is entering from the top. The bullets represent the calculation points. There is cylindrical symmetry around the vertical axis.

Dose calculation points were positioned upstream and downstream from the device, with lateral points implemented further (Fig. 4). From the center of the cylinder outward, points were positioned at 0 cm, 1.15 cm, 1.75 cm, 2.25 cm, and 3.75 cm. This was done identically for distances from the central plane of the port at half thickness (depth 5.65 cm) as well as at distances ±0.25cm and ±0.75cm upstream and downstream from each fat surface (depths 4.25 cm and 4.75 cm upstream and depths 6.55 cm and 7.05 cm downstream). Five planes containing five points each resulted. Homogeneous and heterogeneous planning was conducted involving a total of 300 points for analysis. This incorporated both 6 MV and 18 MV photon energies. Data were analyzed with PBC, AAA, and Monte Carlo algorithms. The zero lateral position at depth 5.65 cm in the water phantom represents the centroid of the device. This point is cylindrically surrounded by titanium, with water assigned both anterior and posterior to it. Points in planes at more shallow depths than that of the central plane will present information on backscatter. Conversely, points in planes deeper than the central plane shed light on forward scattering and attenuation. With the titanium component of the replicate volume present at lateral distances of 0.7 cm to 1.5 cm, it is expected that the most influence will be identified by the dose point assigned at a lateral distance of 1.15 cm. Lateral scattering information was discovered from dose points assigned further out laterally.

The use of AAA and Monte Carlo to accompany the PBC algorithm allows us to quantify accuracy for modeling the correct magnitude of attenuation. This was done by rescaling the 18 vascular access port results using PBC to the ratio of the simulated EGSnrc Monte Carlo system and PBC for the replicate. Rescaling for backscatter and lateral scatter were conducted simultaneously.

## III. RESULTS AND DISCUSSION

For the first part of the study, the results from the PBC treatment planning calculations indicate that vascular access ports with more bulky construction and those with at least partial titanium composition create the largest changes. Some were noted to alter the dose distribution by as much as 5% along the incidence plane. This primarily occurs downstream from the port. Apart from these attenuation changes, little variance was distinguished for backscatter and lateral scatter. The backscatter maximum is found to be within 1%. Between 6 MV and 18 MV data, the results are similar with a maximum difference of 1.1% between the two energies for all data. It was observed that the maximum statistical difference in dose achieved from introducing a vascular access port in the radiation field was found for the Titanium PowerPort model 1708000 at 5.0% for 6 MV energy using PBC. This port contains 1.3 mm of titanium from the anterior face to the posterior base. A review of construction schematics revealed that this port contained the thickest amount of titanium of all 18 models considered. A representative statistical minimum dose change weighted over all calculation points was achieved by the MRI Full Size port model 0605420, which contains no titanium. Almost no change was observed using this port. An average result is exhibited by the Rosenblatt port model 0654970, which has some titanium under its dual septum. It registered about a 2.7% maximum change.

**Figure 5:**
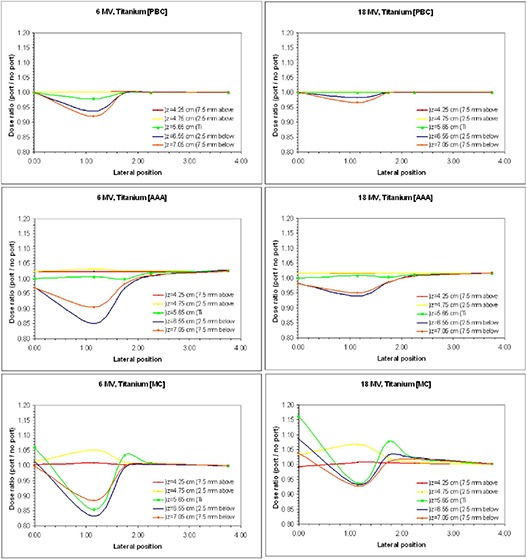
Ratio of dose with the port present to dose in absence of the port for a 1.3 cm Ti replicate of a vascular access port. At left, from the top down are data from algorithms PBC, AAA and EGSnrc Monte Carlo at 6 MV. At right, from the top down are data similarly at 18 MV.

The dose perturbation caused by the presence of the vascular access port was investigated further using alternative algorithms, including Monte Carlo. For simplicity with relative plan comparisons, algorithm calculations were based on a simple design resembling that of the Titanium PowerPort model 1708000. From the previous experiment, it was discerned that this model yielded the most attenuation, due primarily to its thickness of titanium used in its construction. A comparable model of it was expected to present significant changes similarly. Figure 5 shows the rescaled ratios of dose at different positions with the port present to dose when the port has been replaced with water. Within this more complex study, we note vast differences between photon energy, the algorithm employed, planar dose changes at varying distances above (backscattered) and below (attenuated and forward scattered) the device, and lateral scatter behavior specifically detailed at discrete positions from it.

The dose perturbations are due to a combination of attenuation and electron scatter, the latter effect being more important at 18 MV. At 6 MV, there is a dose reduction of 11.6% and 16.8% at 7.5 mm and 2.5 mm downstream from the Ti port. Upstream from the port there is a dose increase of 5% and 0.8% at 2.5 mm and 7.5 mm due to electron backscatter from the Ti. There is a dose increase of up to 3.4% laterally at 2.5 mm away (due to lateral electron scatter) from the Ti port, which reduces to below 0.5% away from the port and away from the plane through the center of the port. At 18 MV, the result is qualitatively similar, although the differences are now 6.9% at 2.5 mm and 7.2% at 7.5 mm downstream from the port and 7% and 0.7% at 2.5 mm and 7.5 mm upstream. The lateral increase is 7.7%, 3.1% and 1.9% in the plane through the center of the port, at 2.5 mm and 7.5 mm downstream from the port, respectively. There is a significant dose increase above and below the center of the port due to forward and backward electron scatter.

Qualitatively, the effects of attenuation directly downstream from the port body were similar in the AAA algorithm when compared to the MC algorithm for both energies, but they are underestimated by the PBC algorithm. The PBC algorithm has been known to overestimate lateral scatter if the pencils are hazy. This volume averaging effect known to be exhibited in Eclipse is a commonality when beam modeling profiles are scanned with a large ionization chamber rather than with a diode or microchamber. The AAA and PBC algorithms were fully commissioned based on Golden Beam Data. This data was acquired at 2.5 mm increments with a Scanditronix‐Wellhöfer (IBA Dosimetry GmbH, Schuarzenbruck, Germany) model CC13‐S ionization chamber. This chamber would likely not present such problems, since it has a very small sensitive volume at only 0.13 cc. With the pencil characteristics in Eclipse based on the scanned profiles, and with both the AAA and PBC algorithms utilizing Golden Beam Data, the modeling component to error in the PBC cannot be determined. However, regardless of the size of the ports, it is likely that the calculated amount of lateral scatter from pencil beams surrounding that region and not traversing the port was the dominant factor for underestimated PBC results. This appears to be a direct consequence of the limitation of the PBC algorithm not being able to account for heterogeneity anisotropically in the entire three‐dimensional vicinity of the interaction site, with the use of photon scatter kernels in multiple lateral directions.^16^ Both the AAA and MC algorithms are capable of conducting such lateral calculations.

Significant effects of electron backscattering, forward scattering and lateral scattering as determined by MC were not evident from either alternative algorithm. Between AAA and PBC, only AAA showed some effects of backscattering but these were minimal at the furthest distance away. All three algorithms agree to an indication of negligible backscattering at the larger distance away from the port, for both energies. As the radiation is directed downstream, there is clearly more forward scattering directly below the center of the high density body, as indicated by the MC results and not modeled by PBC nor AAA. We note that only MC was capable of projecting significant backscatter to be present just outside of the port. This was modeled as attenuation by both AAA and PBC. At 6 MV, the greatest amount of change of backscatter, lateral dose, and attenuation for PBC were 0%, 0%, and −8%. For AAA, these were 3.2%, 2.6%, and −15.0%. The PBC and AAA both show a similar form with more drastic results for 6 MV as compared to 18 MV. At 18 MV, the greatest amount of change of backscatter, lateral dose, and attenuation for PBC were 0%, 0%, and −2.0%. For AAA, these were 1.8%, 1.8%, and −6.0%.

The MC calculations show that more forward and lateral scatter dose contributions are present locally within the cylindrical volume than both PBC and AAA identify. Although within such a device at the centroid, no patient significant information on dose is relevant, this does show the limitation of using the AAA and PBC algorithms. AAA did not prove better than PBC at computing lateral dose for this high‐Z applicator. While PBC hinted toward an increase local to the high density object volume, AAA presented a dose drop. In this regard, AAA proved poorer than the PBC. Nevertheless, dose buildup anterior to the device and attenuation beyond the device were better indicated by AAA. The PBC algorithm was capable of qualitatively calculating the dose reduction due to heterogeneity for each event. The more accurate magnitude of these changes was exhibited by AAA, however. The AAA algorithm was unanimously a better performer to resemble MC for this small, geometrically standard replicate of a commercially available high‐Z vascular access port.

Given the results of the first study, where the PBC algorithm was used for direct calculation of dose for all 18 port models, a relationship can be made to it and the results of the model study. This second study completes the relationship between the PBC algorithm and the Monte Carlo system. A first order correction can be extracted from the relation between the MC and the PBC results for the replicate of the Titanium PowerPort, which can then be applied to the PBC computations for each of the 18 ports. This enables a tabulation of dosimetric effects of each port model. In Table 1, all investigated vascular access ports are presented in ascending order according to the amount of attenuation exhibited. Backscatter and lateral scatter changes are also presented. Two ports ranked differently between the two photon energies, the Ultra Low Profile Port model 0655640 and the X‐Port ISP model 0657500. However, the difference between the two was noted as less than 0.2%.

## IV. CONCLUSIONS

This study details the consequences of having a vascular access port within the field of a radiation therapy beam. Rigorous research on such a broad selection of port devices using advanced treatment planning systems has never been done before. The study includes 18 different ports, representing every model type currently being manufactured from the market leader in vascular access port distribution. The effects of treatment planning simulation were validated by a full Monte Carlo system, EGSnrc with user code DOSRZnrc, on a replicate of the Titanium PowerPort. Comparisons were made to it from results of the Pencil Beam Convolution algorithm and the Analytical Anisotropic Algorithm. Results seen here may now be studied by medical physicists who are presented with a challenge to provide additional information regarding the dosimetric consequences of having a vascular access port in the field or close to the field edge.

Vascular access port involvement caused a change in the distribution of dose in all directions during treatment simulation. However, this was only true for the more bulky designed models and those containing titanium as a major scattering source. Plastic ports did not exhibit significant attenuation or scatter. It is expected that vascular access ports composed of material with higher atomic number than titanium (such as stainless steel) would reveal changes greater than those seen in this study. However, future trends in port manufacturing toward the use of such materials may be less attractive, as is clear from the significant dosimetric effects of titanium observed in this study.

The results of the replicate of the Titanium PowerPort model 1708000 showed a worst‐case scenario dose reduction of 16.8% due to attenuation. The magnitude of this result is similar to measured results from Bagne et al.^(1)^ involving a small selection of ports, where one contained steel. Depending on the location of interest, increases and decreases in dose were noted. Upstream from the port, there was a dose increase of 5.0% due to electron backscatter from the Ti at 2.5 mm. There was a dose increase of up to 3.4% laterally at the same distance away (due to lateral electron scatter) from the Ti port. At 18 MV, the results were qualitatively similar, although the differences became 7.2% at 7.5 mm downstream from the port, 7.0% upstream at 2.5 mm, and laterally dose increased by 7.7% at 2.5 mm. There is a significant dose increase above and below the center of the port due to forward and backward electron scatter.

These data conclude that port placement and port selection should be carefully considered prior to patient application. If circumstances are foreseen where the port placement might impact treatment delivery, the use of a plastic port is suggested. However, clinical impact on dose delivery may also be aided by port placement alternatives, such as repositioning or removal. One should always identify the manufacturer and model of port being used. The design construction should also be evaluated. By intercomparison to models presented here, it may be observed how vascular access ports affect high energy radiation beams. Such information may enable greater clinical understanding and more educated judgment on the accuracy of treatment planning results. Although some treatment planning systems provide more accurate dosimetry than others, we have shown here that it is possible to determine an adequate dose effect magnitude from two common treatment planning system algorithms in comparison to Monte Carlo. Further, it may now be possible to intercompare these results to dosimetry observed clinically to aid in the decision and discussion of dosimetry when patients are involved.

## ACKNOWLEDGEMENTS

We are grateful to have been given the opportunity to study each of the access ports manufactured by Bard Access Systems, Inc. Thus, we applaud the initiative of the company to supplement the pursuit for greater understanding of access port science, which enables better clinical choices in medicine. This work was supported by the issuance of Bard Access Systems, Inc. Grant # 7019.

We would also like to thank Clinical Specialist JoAnn Smith BSN, CWOCN, CRNI for her support and interest in research and development, as well as her initiative in directing the initial contact with Bard Access Systems, Inc.
